# Deposition of Heavy Metals in Patients with Deep Venous Thrombosis and Healthy Individuals: A Case–Control Study with Laser-Induced Breakdown Spectroscopic Analysis of Nail Edges

**DOI:** 10.3390/jcm15051786

**Published:** 2026-02-27

**Authors:** Lutfi Çagatay Onar, Gunduz Yumun, Havva Nur Alparslan Yumun, Muhammed Habib Onen, Didem Melis Oztas, Murat Ugurlucan

**Affiliations:** 1 Department of Cardiovascular Surgery, Republic of Turkey Ministry of Health, Dr. Ismail Fehmi Cumalioglu City Hospital, Tekirdag 59020, Turkey; 2 Department of General Surgery, Republic of Turkey Ministry of Health, Dr. Ismail Fehmi Cumalioglu City Hospital, Tekirdag 59020, Turkey; 3 Council of Forensic Medicine, Istanbul 34196, Turkey; 4 Department of Cardiovascular Surgery, Vadistanbul Liv Hospital, Istanbul 34475, Turkey

**Keywords:** LIBS, DVT, elements magnesium

## Abstract

**Background**: Deep vein thrombosis (DVT) is one of the most common cardiovascular diseases and is especially prevalent in areas with environmental pollution. Bioaccumulation of toxic heavy metals may lead to deterioration of homeostasis with cellular change, endothelial dysfunction, DNA impairment and cellular signaling. The reason for this is usually the accumulation of thrombogenic toxins in the body as a result of long-term exposure or a lack of regulatory gene expression. In this study, we aimed to measure the minerals that potentially accumulate in the nail. The measurement method was laser-induced breakdown spectroscopy (LIBS), which is a form of atomic emission spectroscopy. It uses a highly energetic laser source to form a plasma of excited atoms emitting light of characteristic wavelengths. It provides accurate quantification and reveals the relationship between tissue accumulation of toxic heavy metals and DVT formation. **Methods**: Between January 2020 and December 2021, 100 patients diagnosed with lower-extremity deep vein thrombosis were screened in a single tertiary healthcare center. Among them, 50 patients who met the eligibility criteria and consented to participate were included in the study. An additional 50 age-matched healthy volunteers were enrolled as controls. Demographic and clinical characteristics were recorded. Nail samples were obtained from each participant, and elemental emission intensities were quantitatively analyzed using laser-induced breakdown spectroscopy (LIBS). **Results**: No difference in clinical characteristics was detected between the groups. While iron, calcium and silicon were found to be high in DVT patients, magnesium was found to be low. Regarding the magnesium emission, ROC analysis showed 76–90% specificity and 69–82% sensitivity, respectively. **Conclusions**: LIBS is a useful method because it is easy to use and can be used with a small sample. According to the results of our study, information about the pathogenesis of DVT was obtained through nail analysis. Therefore, we believe that LIBS analysis is a method that may be useful in determining the causes and predisposing factors for DVT.

## 1. Introduction

Venous thromboembolism is one of the most common and costly diseases of the cardiovascular system. It is related to a wide spectrum of factors such as hereditary pro-thrombotic conditions, endothelial dysfunction, blood viscosity and stasis, vascular trauma and inflammatory stimuli. It is also complicated by different clinical conditions that later determine the clinical endpoint or recurrence. The complications of deep venous thrombosis (DVT) may be life-threatening. The treatment strategies may require long-term or sometimes life-long anticoagulation medication and external compression [1]. Hypercoagulation is a hallmark of thromboembolism, which can manifest as DVT or pulmonary embolism (PE). Trauma, immobilization, and malignancy are other risk factors [2]. Once environmental toxins and heavy metals reach the blood circulation, they permeate into capillaries, endothelial cells and pericytes. This may lead to endothelial dysfunction (ED) and can trigger a thrombotic cascade [3]. At that point, tissue bioaccumulation of heavy metals begins. We know that the deficiency of vitamin D and magnesium is associated with venous thrombosis [4,5], but there are only a few studies that have investigated the excessive tissue bioaccumulation of heavy metals and toxins as a potential initiator or predisposing factor of venous thromboembolism. One of the most probable potential triggering factors is the gathering of pro-inflammatory cytokines due to the bioaccumulation of heavy metals. The NF-K B signaling pathway is responsible for the pro-inflammatory and coagulation cascades. It is strongly believed that the NF-K B signaling pathway is also responsible for other cardiovascular emergency conditions such as acute coronary syndrome, pulmonary embolism and stroke.

Laser-induced breakdown spectroscopy (LIBS) is a technology for fast remote chemical analysis based on the excitation of plasma on the surface of a sample by a short laser pulse and the study of its spectral composition. LIBS technology is applicable to many samples, including metals, semiconductors, glasses, and biological tissues [6,7]. LIBS analysis provides exact quantification of trace elements in human tissues with high sensitivity [8].

Nail tissue represents a cumulative biological matrix that reflects long-term trace element exposure due to its slow growth rate and metabolic stability. Unlike serum biomarkers, which may fluctuate with acute physiological changes, nail analysis provides retrospective information regarding chronic elemental status. In the context of thrombotic disorders such as DVT, chronic trace element imbalance may contribute to endothelial dysfunction, platelet activation, and vascular remodeling. Therefore, identifying elemental alterations through a non-invasive technique such as LIBS may offer complementary insights for cardiovascular risk assessment and long-term thrombotic susceptibility. The aim of this study is to analyze whether there is heavy metal accumulation or trace element deficiency in patients with lower-extremity DVT by performing nail analysis using the LIBS method.

## 2. Materials and Methods

Participants were consecutively recruited from the Cardiovascular Surgery Outpatient Clinic of Tekirdağ Dr. İsmail Fehmi Cumalıoğlu City Hospital between January 2020 and December 2021. All eligible patients presenting during the study period were screened according to predefined inclusion and exclusion criteria.

Individuals were categorized into two groups: a patient group (50) of those with DVT and a healthy control group (50) of those without DVT. A total of 25 women, 10 (20%) in the DVT group and 15 (30%) in the control group, participated in the study. Control participants were additionally screened for previous thromboembolic events, inherited thrombophilia, chronic dermatologic nail disorders, and use of medications or supplements affecting mineral metabolism. The demographic, clinical, and laboratory characteristics of the patients and control subjects were obtained from the patient information folders. In addition to the routine blood tests, serum TNF-ɑ, IL-6 and IL-8 were also analyzed.

Age and sex distributions were recorded and compared between groups, as structural characteristics of the nail plate may vary across life stages and between sexes.

The diagnosis of DVT was established by clinical examination and Doppler USG following the ESC diagnosis and treatment guide recommendations [9].

Inclusion criteria: Patients and healthy subjects who were older than 18 years, who had undergone routine physical examinations and laboratory tests, and who accepted to participate in the study were included. All patients in the study had undergone a lower-extremity Doppler ultrasound in the last year. Patients and healthy individuals (control group) were added to the study until the total number of participants reached 100.

Exclusion criteria: Patients who did not give their consent to take part in the study, who were under 18 years old, who had a large necrotic wound or acute arterial ischemia, who had serious metabolic diseases or malignancies, who had undergone major surgery, or who had major trauma were excluded from the study.

Nail samples: Nail samples were collected from participants in both the DVT and control groups from the distal free edge of the great toenail to ensure standardization and minimize external contamination. After clipping, samples were placed in separate plastic containers and stored at 20 ± 3 °C until spectral analysis. Prior to LIBS measurements, samples were cleaned according to the International Atomic Energy Agency (IAEA) recommended washing protocol for trace element analysis [10], using sequential acetone and deionized water rinses; each cleaning step lasted approximately 10 min, and ultrasonication was not applied to avoid structural alteration of the nail matrix.

**Figure 1:**
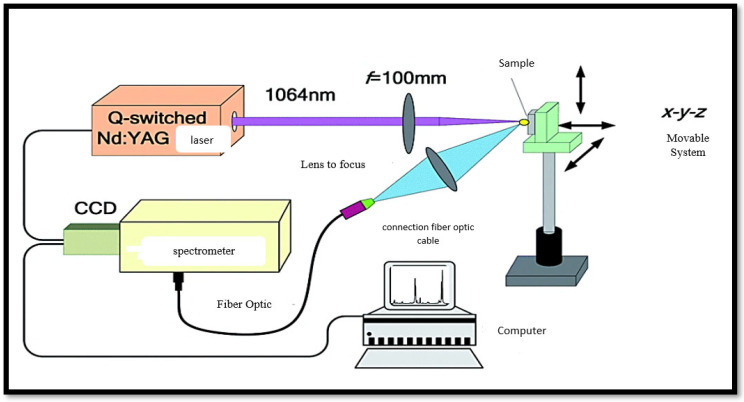
A simple model of laser-induced breakdown spectroscopy [11].

Experimental setup: LIBS is an atomic emission spectroscopy technique for solid, liquid, and gas samples based on the generation of plasma at high temperatures by focusing a high-intensity pulsed laser source on the surface to be analyzed. The components used are given in Figure 1. The experiment was performed in normal atmospheric conditions. In this experiment, each spectrum of the sample was recorded 5 times and averaged to improve the stability of the spectral intensity measurements. The spectra were taken in different positions of the nail with the following standards: pulse energy: 60 mJ; pulse duration: 8 ns; repetition rate: 5 Hz; gate delay: 1.5 µs; integration time: 20 µs.

To identify elements, LIBS spectra were compared to those from the National Institute of Standards and Technology Electronic (NIST) Database [11]. The LIBS spectral analyses were recorded over a wavelength range from 225 nm to 925 nm for all samples.

Statistical analysis: Data were analyzed using SPSS, version 18.0 (SPSS, Inc., Chicago, IL, USA). The differences in the proportions of cases and controls responding to each question were tested by the Chi-square test. The Mann–Whitney U test was used to compare all quantitative variables between cases and controls. Results are presented as percentages and the corresponding *p*-value, and as medians and interquartile range (IQR). The statistical relevance of DVT and the levels of TNF-ɑ, IL-6 and IL-8 were analyzed using the Kruskal–Wallis Test.

## 3. Results

The clinical features and nail LIBS analysis results of a total of 100 people in the patient and control groups were compared. The clinical characteristics of the patient and control groups are shown in Table 1.

For each element, five laser shots were fired, and the average of the radiation amounts was recorded. In the DVT group, the amount of radiation in the known element-specific spectra for iron (Fe 259.91), silicon (Si 288.17, Si 243.53, and Si 251.65), and calcium (Ca 422.70) was found to be higher than in the control group. In the magnesium analysis, a lower amount of radiation was observed in the three known different Mg light spectrums (Mg 285.20, Mg 279.53, and Mg 518.49) than in the DVT group (Table 2). Among the analyzed spectral lines, Mg 518.49 demonstrated the highest diagnostic performance (AUC = 0.907), indicating excellent discrimination between DVT patients and controls. An attempt was made to determine spectral threshold values by performing ROC analysis.

**Figure 2:**
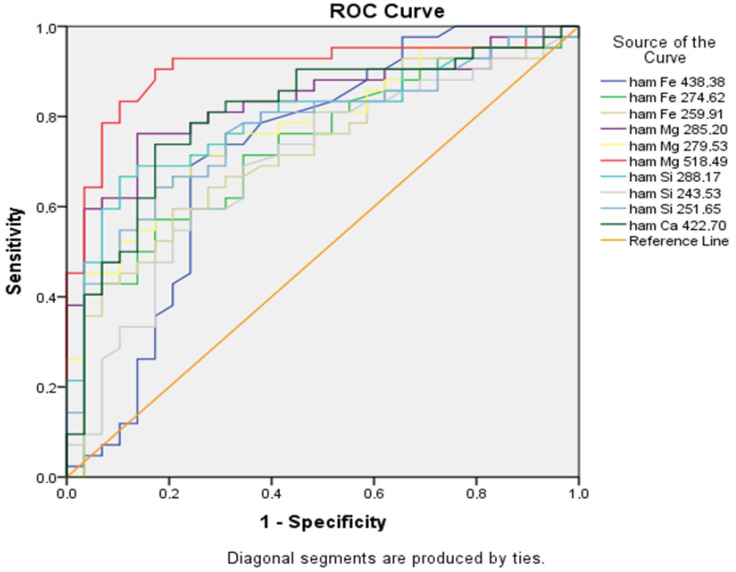
The relationship between sensitivity and specificity for deep vein thrombosis with spectral reflection amounts representing the elements in the ROC curve analysis. Element-under-the-curve measurements correlate with sensitivity and specificity as indicators of disease.

In the quantitative LIBS analysis of nail samples, a ROC curve test was performed for specificity and sensitivity analysis according to the spectral amounts of the elements. In the examination performed within the 95% confidence interval, the spectral cut-off levels, specificity and sensitivity levels of the elements were determined and are shown in detail in Table 3. Magnesium (Mg 518.49) has the largest curve area. It also has the highest sensitivity (90.5%) and specificity (82.7%) (Figure 2).

Distinct differences were observed in the LIBS spectral profiles of healthy and pathological nails, particularly in calcium-related emission intensities; sodium and potassium lines were detected, but due to insufficient signal stability and reproducibility, they were not included in the quantitative and ROC analyses.

## 4. Discussion

In this study, the toenails of patients who had DVT in the last 12 months and individuals in the control group were analyzed with the LIBS method to measure the amount of elements in their toenails. The results obtained were compared between groups consisting of individuals with DVT and controls. The nail tissue levels of iron, silicon and calcium were found to be higher in the DVT patients compared to controls. The levels of magnesium were lower in the DVT group, whereas lead, which has been shown to have toxic effects in previous studies, was found to be at similar levels in both groups in the nail analysis. The levels of zinc and copper, which are expected to cause pathological thrombogenic events in the absence of deficiency in the organism, were at similar levels in the groups according to our measurements.

The serum levels of TNF-ɑ, IL-6 and IL-8 were found to be higher in the DVT group compared to the healthy individual group.

LIBS is an optical emission technique that produces a spark directly on the sample using a high-power pulsed laser beam. Material identification is done using atomic emission spectroscopy. Concentrations are determined using the relative light intensity. Many elements can be analyzed with LIBS. LIBS can be applied to solid, liquid and gas samples. Another important feature of LIBS is that it is sensitive enough to detect elements in the expected range. LIBS first appeared in 1962 as atomic emission spectroscopy, and it is still being studied intensively today [6,12].

LIBS analyses have been applied in biological investigations with studies in forensic medicine. Nowadays, it is used in the fields of cancer, food and tissue sample analysis [13].

The clinical conditions most closely associated with DVT are fundamentally related to the elements of Virchow’s Triad; these include surgery or trauma, malignancy, prolonged immobility, pregnancy, congestive heart failure, varicose veins, obesity, advancing age, and a history of DVT [14]. Previous studies have found relationships between environmental pollution and thromboembolism [5], and it has been shown that long-term exposure to polluted environments causes various cardiovascular diseases [15]. In this study, although there was no occupational exposure, we examined chronic accumulations or element deficiencies that may be caused by the living area or water.

Magnesium is known to modulate endothelial nitric oxide synthesis, regulate calcium channel activity, and reduce platelet aggregation [16]. Experimental and clinical studies suggest that magnesium deficiency may promote vascular inflammation, oxidative stress, and hypercoagulability. Altered intracellular magnesium levels may influence thrombus formation through endothelial dysfunction and platelet activation pathways [16]. However, whether nail magnesium intensity directly reflects systemic bioavailable magnesium remains uncertain and requires mechanistic validation in prospective studies.

In addition to its general vascular regulatory functions, alterations in systemic magnesium levels have been associated with changes in inflammatory signaling pathways, oxidative stress balance, and endothelial cell survival mechanisms. Experimental evidence suggests that magnesium deficiency may enhance pro-inflammatory cytokine production, increase intracellular calcium overload, and promote a pro-thrombotic phenotype through platelet hyperreactivity and impaired nitric oxide bioavailability. Conversely, physiological magnesium concentrations appear to exert protective effects by modulating oxidative stress and maintaining endothelial integrity. These findings provide a plausible biological framework linking altered magnesium-related spectral intensity in nail tissue to systemic vascular dysregulation, although causality cannot be inferred from the present observational design [17].

There are studies showing that it may have protective effects on cardiovascular diseases [18]. However, a prospective cohort study did not show a relationship between serum magnesium and VTE [16]. In this study, after laser shots were applied to the nails, radiation in the spectrum specific to magnesium at three different wavelengths was analyzed. Magnesium amounts were found to be statistically lower compared to the control group. In particular, the amount of radiation in the Mg 518.49 spectrum was evaluated as a highly sensitive marker for DVT. The sodium and potassium spectral lines were not analyzed quantitatively because their signal stability was inadequate for reliable reproducibility.

Previously, it was shown that silica nanoparticles shortened coagulation time in APTT and PT tests and increased the activation of factor X [19]. In another study, Feng and colleagues showed that silica nanoparticles can cause endothelial damage [20]. In this study, a conclusion was reached in accordance with the literature, and according to the radiation detected in the nail, the amount of silicon was found to be higher in the DVT group than in the control group.

Iron deficiency is an underestimated thromboembolic risk factor. Although secondary thrombocytosis occurring with IDA is generally thought to be harmless, there is accumulating evidence that high platelet counts, especially in the setting of iron deficiency, may lead to increased thromboembolic risk in both arterial and venous systems [21]. It was shown that both iron deficiency and excess have been associated with an increased risk of developing thromboembolic events [22]. In this study, we see that iron accumulation is high in the nails of patients who have experienced DVT. The radiation indicating iron was high in all three wavelengths (Fe 438.38; Fe 274.62; Fe 259.91).

The Pb exposure can be caused by atmospheric pollution [23]. In another study, almost all lead accumulation occurs in erythrocytes [24]. As a result, the coagulation process is triggered, and the tendency to thrombosis increases [25]. In this study, no significant difference in Pb was found between the two groups. Therefore, we can say that lead shows its toxic effect through erythrocyte accumulation rather than accumulation.

In addition to air pollution, several pollutants can be absorbed in the diet [26]. Excessive consumption of seafood or sometimes even medications can cause accumulation of some elements and heavy metals in the body [27].

From a clinical perspective, nail LIBS analysis is not intended to replace established diagnostic methods but may serve as a complementary non-invasive screening approach in cardiovascular research settings. In podiatric and vascular clinics, standardized nail trace element assessment may provide additional information regarding chronic metabolic or thrombotic risk patterns. Nevertheless, routine clinical implementation requires multicenter validation and cost-effectiveness evaluation.

### Study Limitations

This study has several limitations that should be acknowledged. First, detailed dietary habits and micronutrient intake of the participants could not be comprehensively documented; therefore, the potential contribution of nutritional exposure to elemental accumulation could not be evaluated. Second, environmental exposure characteristics such as residential proximity to pollution sources and duration of exposure were not systematically assessed, precluding analysis of environmental burden and tissue accumulation relationships. Third, blood element measurements were not performed to correlate systemic levels with nail concentrations, as most patients were evaluated during the subacute or chronic phase of DVT, when circulating levels may not accurately reflect long-term tissue deposition. Although medications influencing mineral metabolism were excluded in the control group, subgroup analyses according to specific drug classes were not performed. Therefore, residual confounding related to pharmacological factors cannot be entirely excluded.

## 5. Conclusions

In conclusion, this study demonstrates measurable differences in selected LIBS-derived trace element intensities between patients with DVT and control subjects, particularly regarding magnesium. These findings suggest a potential association between chronic elemental imbalance and thrombotic conditions. However, given the observational design and limited sample size, the results should be interpreted as hypothesis-generating. Further prospective studies with standardized protocols are required to clarify biological mechanisms and clinical applicability.

## Figures and Tables

**Table 1:** Some qualitative variables of cases and controls.

Patient Characteristics	Patients (50)	Controls (50)	*p*
Gender (F)	20 (40)	17 (34)	0.339
Age	65.5 ± 9.18	67.2 ± 9.87	0.364
Smokers	13 (26)	6 (12)	0.252
Drug Use	21 (42)	25 (50)	0.274
Alcohol Consumption	7 (14%)	9 (18%)	0.100

**Table 2:** Comparison of element-specific spectrum radiation amounts in nails analyzed with the LIBS method between two groups.

Element (nm)	DVT Group (Mean ± SD)	Control Group (Mean ± SD)	*p*-Value
O_2_ 777.29	5189.2 ± 1262.8	5322.2 ± 562.8	0.582
Fe 259.91	1017.2 ± 562.8	697.5 ± 506.2	0.015
Mg 285.20	1252.7 ± 386.7	2324.7 ± 1067.9	<0.001
Mg 279.53	6772.9 ± 4358.5	13,605.4 ± 7605.4	<0.001
Mg 518.49	223.9 ± 30.4	335.6 ± 104.2	<0.001
Si 288.17	1277.7 ± 793.5	615.3 ± 278.6	<0.001
Si 243.53	654.2 ± 62.2	618.2 ± 44.7	0.006
Si 251.65	929.7 ± 363.6	643.0 ± 161.5	0.001
Ca 422.70	2332.9 ± 1049.2	1296.8 ± 690.2	<0.001

**Table 3:** Interaction of ROC analysis and element analysis with DVT and cut-off values.

Element (nm)	AUC	Cut-Off	Sensitivity (%)	Specificity (%)	*p*-Value	95% CI (Lower–Upper)
Fe 438.38	0.723	179.5	73.8	69.0	0.001	0.592–0.854
Fe 274.62	0.730	472.5	71.4	65.5	0.001	0.612–0.848
Fe 259.91	0.723	636.5	69.0	62.1	0.001	0.604–0.842
Mg 285.20	0.833	1367	81.0	72.4	<0.001	0.738–0.927
Mg 279.53	0.781	7926	76.2	69.0	<0.001	0.675–0.887
Mg 518.49	0.907	244.5	90.5	82.7	<0.001	0.834–0.981
Si 288.17	0.799	686.5	73.8	72.4	<0.001	0.696–0.903
Si 243.53	0.693	620.5	73.8	58.6	0.006	0.567–0.818
Si 251.65	0.770	641	76.2	69.0	<0.001	0.660–0.880
Ca 422.70	0.806	1453	81.0	72.4	<0.001	0.701–0.912

## Data Availability

No new data were created or analyzed in this study.
